# Supplementary material for: Evolution of the Output–Workforce Relationship in Primary Care Facilities in China from 2009 to 2017
Source: Int J Environ Res Public Health. 2020 Apr 27;17(9):3043. doi: 10.3390/ijerph17093043 (PMC7246558; doi:10.3390/ijerph17093043)
Supplement: Supplementary file 1 [file ijerph-17-03043-s001.pdf]

Table 1. Characteristics of the primary care facilities.

Characteristics	Primary Care Facilities (n=785)
Type	
Township health centre (%)	420 (53.5)
Community health centre/station (%)	365 (46.5)
Urbanity	
Urban (%)	370 (47.1)
Rural (%)	415 (52.9)
Average number of workforce per facility (SD)	33.5 (30.0)
Average number of beds per facility (SD)	31.9 (40.2)
Average service building area in 100 m^2 per facility (SD)	21.1 (23.5)
Average number of equipment over 10,000 yuan per facility (SD)	14.5 (15.8)

notes: a. SD means standard deviation. b. Data for human resource, beds, infrastructure and equipment are for 2017.

Table 2. Types of basic public health services.

Services	2017	2015	2011	2009	Before 2009
Establishing health records	√	√	√	√	√
Health education	√	√	√	√	√
Health management for children (0–6 years old)[1]	√	√	√	√	√
Maternal health management	√	√	√	√	√
Vaccination	√	√	√	√	√
Reporting and handling of infectious diseases and public health emergencies[2]	√	√	√	√	√
Health management for the elderly	√	√	√	√	
Health management for patients with hypertension	√	√	√	√	
Health management for patients with type 2 diabetes	√	√	√	√	
Management for patients with severe mental illness	√	√	√	√	
Supervising and managing health and family planning[3]	√	√	√		
Health management for patients with tuberculosis	√	√			
Traditional Chinese medicine	√	√			
Providing free contraceptives	√				
Promoting health literacy	√				

1. Health management for children only include those aged 0–36 months before 2011. 2. Reporting and handling public health emergencies were excluded before 2011. 3. Supervising and managing family planning were excluded before 2017.

Table 3. Number of primary care facilities with data for output-workforce relationship.

Years of Data	Clinical Care		Public Health Services	
	Number	Percentage	Number	Percentage
9	384	48.92%	332	42.29%
8	39	4.97%	62	7.90%
7	42	5.35%	63	8.03%
6	28	3.57%	32	4.08%
5	34	4.33%	37	4.71%
4	23	2.93%	24	3.06%
3	22	2.80%	21	2.68%
2	17	2.17%	0	0.00%
less than 2*	196	24.97%	214	27.26%
Total	785	100.00%	785	100.00%

*Notes: Facilities only had data for less than 2 years were because of the following reasons: a. Some facilities just established for one or two years; b. There were missing data for output because of some technical reason. For example, old data were missing when the Health Statistics Reporting system was updated. c. There were missing data for number of workforce. For public health services, some facilities did not have written documents for the number of staffs and had difficulties in reporting the number in our survey.

Table 4. Number of primary care facilities with missing data for characteristic variables.

**VariablesA2:G38**	**Clinical Care**			**Public Health Services**		
		**Number**	**Percentage**		**Number**	**Percentage**
Time-stable characterisitcs						
GRP per capita		24	4.07%		23	4.03%
region		0	0.00%		0	0.00%
urban		0	0.00%		0	0.00%
landform		3	0.51%		3	0.53%
service radius		9	1.53%		7	1.23%
	**Year**	**Number**	**Percentage**	**Year**	**Number**	**Percentage**
Time-dependent characterisitcs						
Human resource	2009	105	17.83%	2009	103	18.04%
	2010	105	17.83%	2010	104	18.21%
	2011	71	12.05%	2011	68	11.91%
	2012	55	9.34%	2012	52	9.11%
	2013	22	3.74%	2013	20	3.50%
	2014	11	1.87%	2014	9	1.58%
	2015	6	1.02%	2015	4	0.70%
	2016	3	0.51%	2016	4	0.70%
	2017	17	2.89%	2017	15	2.63%
Beds	2009	110	18.68%	2009	108	18.91%
	2010	106	18.00%	2010	105	18.39%
	2011	72	12.22%	2011	70	12.26%
	2012	61	10.36%	2012	58	10.16%
	2013	37	6.28%	2013	35	6.13%
	2014	29	4.92%	2014	24	4.20%
	2015	23	3.90%	2015	16	2.80%
	2016	21	3.57%	2016	17	2.98%
	2017	30	5.09%	2017	24	4.20%
Infrastructure	2009	108	18.34%	2009	106	18.56%
	2010	110	18.68%	2010	109	19.09%
	2011	76	12.90%	2011	74	12.96%
	2012	60	10.19%	2012	58	10.16%
	2013	29	4.92%	2013	27	4.73%
	2014	17	2.89%	2014	15	2.63%
	2015	10	1.70%	2015	6	1.05%
	2016	7	1.19%	2016	7	1.23%
	2017	21	3.57%	2017	18	3.15%
Equipment	2009	124	21.05%	2009	119	20.84%
	2010	127	21.56%	2010	123	21.54%
	2011	87	14.77%	2011	82	14.36%
	2012	64	10.87%	2012	61	10.68%
	2013	32	5.43%	2013	29	5.08%
	2014	18	3.06%	2014	15	2.63%
	2015	18	3.06%	2015	14	2.45%
	2016	12	2.04%	2016	12	2.10%
	2017	22	3.74%	2017	19	3.33%

Table 5. STROBE checklist.

	Item No	Recommendation	Section	Paragraph
Title and abstract	1	(*a*) Indicate the study's design with a commonly used term in the title or the abstract	Abstract	2
		(*b*) Provide in the abstract an informative and balanced summary of what was done and what was found	Abstract	2,3,4
**Introduction**				
Background/rationale	2	Explain the scientific background and rationale for the investigation being reported	Introduction	4
Objectives	3	State specific objectives, including any prespecified hypotheses	Introduction	4
**Methods**				
Study design	4	Present key elements of study design early in the paper	Section 2.1	1
Setting	5	Describe the setting, locations, and relevant dates, including periods of recruitment, exposure, follow-up, and data collection	Section 2.1	1
Participants	6	(*a*) *Cohort study*—Give the eligibility criteria, and the sources and methods of selection of participants. Describe methods of follow-up	N/A	N/A
		Case-control study—Give the eligibility criteria, and the sources and methods of case ascertainment and control selection. Give the rationale for the choice of cases and controls	N/A	N/A
		Cross-sectional study—Give the eligibility criteria, and the sources and methods of selection of participants	Section 2.1	1
		(*b*) *Cohort study*—For matched studies, give matching criteria and number of exposed and unexposed	N/A	N/A
		Case-control study—For matched studies, give matching criteria and the number of controls per case	N/A	N/A
Variables	7	Clearly define all outcomes, exposures, predictors, potential confounders, and effect modifiers. Give diagnostic criteria, if applicable	Section 2.2<br>Section 2.3<br>Section 2.4	3<br>4<br>1
Data sources/ measurement	8*	For each variable of interest, give sources of data and details of methods of assessment (measurement). Describe comparability of assessment methods if there is more than one group	Section 2.2<br>Section 2.3<br>Section 2.4	1,2,3<br>1,2,3,4<br>1
Bias	9	Describe any efforts to address potential sources of bias	Section 2.2	1
Study size	10	Explain how the study size was arrived at	Section 2.1	1
Quantitative variables	11	Explain how quantitative variables were handled in the analyses. If applicable, describe which groupings were chosen and why	Section 2.4<br>Section 2.5	1<br>1
Statistical methods	12	(*a*) Describe all statistical methods, including those used to control for confounding	Section 2.5	1
		(*b*) Describe any methods used to examine subgroups and interactions	N/A	N/A
		(*c*) Explain how missing data were addressed	Section 2.5	1
		(*d*) *Cohort study*—If applicable, explain how loss to follow-up was addressed	N/A	N/A
		Case-control study—If applicable, explain how matching of cases and controls was addressed	N/A	N/A
		Cross-sectional study—If applicable, describe analytical methods taking account of sampling strategy	N/A	N/A
		(*e*) Describe any sensitivity analyses	Section 2.5	1
**Results**				
Participants	13*	(a) Report numbers of individuals at each stage of study—eg numbers potentially eligible, examined for eligibility, confirmed eligible, included in the study, completing follow-up, and analysed	Section 2.1<br>Section 2.5	1<br>1
		(b) Give reasons for non-participation at each stage	N/A	N/A
		(c) Consider use of a flow diagram	N/A	N/A

Descriptive data	14*	(a) Give characteristics of study participants (eg demographic, clinical, social) and information on exposures and potential confounders	Section 3	1
		(b) Indicate number of participants with missing data for each variable of interest	Section 3.4<br>Section 3.5	1<br>1
Outcome data	15*	*Cohort study*—Report numbers of outcome events or summary measures over time	N/A	N/A
		Case-control study—Report numbers in each exposure category, or summary measures of exposure	N/A	N/A
		Cross-sectional study—Report numbers of outcome events or summary measures	N/A	N/A
Main results	16	(*a*) Give unadjusted estimates and, if applicable, confounder-adjusted estimates and their precision (eg, 95% confidence interval). Make clear which confounders were adjusted for and why they were included	N/A	N/A
		(*b*) Report category boundaries when continuous variables were categorized	N/A	N/A
		(*c*) If relevant, consider translating estimates of relative risk into absolute risk for a meaningful time period	N/A	N/A
Other analyses	17	Report other analyses done—eg analyses of subgroups and interactions, and sensitivity analyses	section 3.4.1<br>section 3.4.2	1<br>1
Discussion				
Key results	18	Summarise key results with reference to study objectives	Discussion	1
Limitations	19	Discuss limitations of the study, taking into account sources of potential bias or imprecision. Discuss both direction and magnitude of any potential bias	Discussion	8
Interpretation	20	Give a cautious overall interpretation of results considering objectives, limitations, multiplicity of analyses, results from similar studies, and other relevant evidence	Discussion	7
Generalisability	21	Discuss the generalisability (external validity) of the study results	Discussion	9
Other information				
Funding	22	Give the source of funding and the role of the funders for the present study and, if applicable, for the original study on which the present article is based	Funding	1

*Give information separately for cases and controls in case-control studies and, if applicable, for exposed and unexposed groups in cohort and cross-sectional studies. *Give information separately for cases and controls in case-control studies and, if applicable, for exposed and unexposed groups in cohort and cross-sectional studies.
